# Home is where your heat is: local conditions forge a fish’s temperature tolerance

**DOI:** 10.1093/conphys/coaf021

**Published:** 2025-04-05

**Authors:** Zoe Storm

**Affiliations:** College of Science and Engineering, James Cook University, Townsville 4810, Australia

Can your home make you tougher? For one of North America’s most well-known cold-water fish, it just might. A recent study by Trent University researchers (Stewart et al., 2024) found that a brook trout’s (*Salvelinus fontinalis*) ‘home’ plays an important role in how it copes with heat: trout have a greater tolerance to elevated water temperatures if they have been exposed to warmer stream temperatures in the weeks leading up to the heat. As climate change heats up waters across the globe, these past experiences could be the key to their survival (Fig. 1).

**Figure 1:**
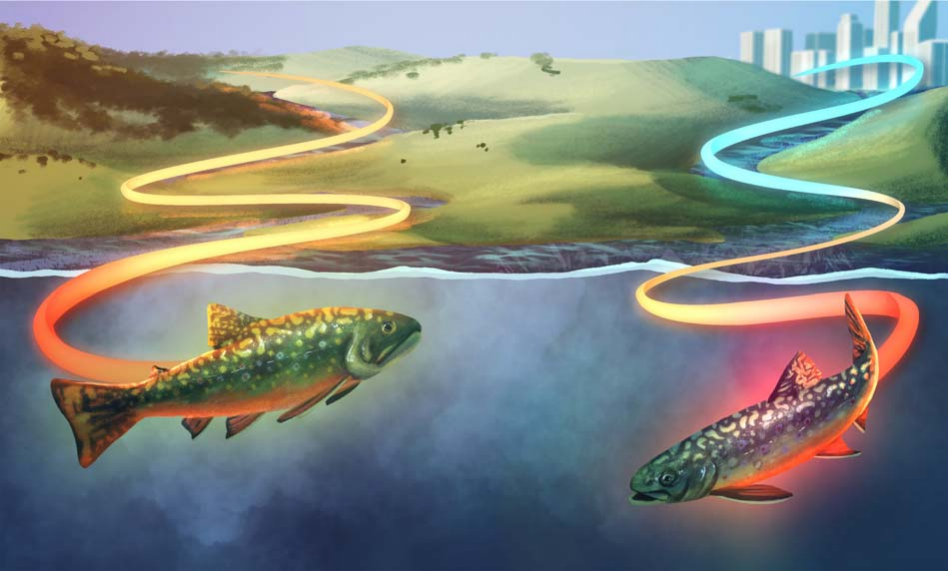
Illustration: Kaitlin Barham (kaitlin.barham@student.uq.edu.au)

The idea that our homes shape who we are is nothing new. In nature, survival depends on species evolving over generations to adapt to the conditions around them. However, climate change is outpacing species’ genetic adaptation, creating a selection pressure for those genotypes that can acclimate rapidly. This raises an important question: does an animal’s environmental history influence its ability to cope with rapid change? Understanding how past environments shape future survival could be key to conserving species in a changing world.

Brook trout inhabit diverse freshwater habitats, from small spring-fed streams to the Great Lakes. In areas with fragmented rivers or dams, trout populations become genetically distinct as individuals adapt to their local conditions. While heat tolerance is partly inherited, environmental experience also plays a role. Indeed, lab studies have revealed that trout from different regions have varying heat tolerances, but the specific factors driving this variation remained unclear.

To explore what influences brook trout’s heat tolerance, Erin Stewart and her team studied 20 populations of brook trout across Ontario, Canada. They selected sites that had different water temperatures and populations that were largely genetically distinct. The researchers captured trout from each location and placed them in tanks near their streams. They gradually heated the water in the tanks to determine the critical thermal maximum of the fish (CT_max_) — the highest temperature that trout could withstand before they became unable to keep their body position upright. By comparing the CT_max_ of each population to the highest local water temperature recorded between May and October, they calculated a thermal safety margin, which represents the buffer between a population’s heat tolerance and the peak temperatures in its environment. Thermal safety margins help identify which populations are most at risk and inform conservation strategies, with a larger thermal safety margin suggesting resilience and a smaller one signalling vulnerability. Researchers also recorded and calculated stream temperatures at each site in the 30 days before CT_max_ measurements to determine the effects of acclimation temperature on heat tolerance.

The researchers found that CT_max_ ranged from 27.41 to 30.46°C between sites and that stream temperatures varied widely between locations. The average stream temperature over the 30 days prior to measurements explained most of the differences in heat tolerance between sites, where the CT_max_ of trout increased by on average 0.23°C per 1°C increase in the 30-day acclimation temperature. Safety margins also differed drastically, with some populations having small buffers (as low as 0.51°C) while others had a substantial cushion (up to 15.51°C). Trout in streams near cities, farms or fed by lake water had the smallest safety margins, making them most vulnerable to warming.

The key takeaway? Where a trout lives is critical to its heat tolerance and resilience to climate change. Conservation efforts should therefore focus on understanding habitat-specific conditions and on developing local, population-based management strategies to support the survival of different brook trout populations.

## Notes

**Editor:** Ashleigh Donaldson
